# Sedation in French intensive care units: a survey of clinical practice

**DOI:** 10.1186/2110-5820-3-24

**Published:** 2013-08-09

**Keywords:** Sedation, Midazolam, Propofol, Opioids, Intensive care unit, Mechanical ventilation, Practice survey, Oversedation

## Abstract

**Background:**

Sedation is used frequently for patients in intensive care units who require mechanical ventilation, but oversedation is one of the main side effects. Different strategies have been proposed to prevent oversedation. The extent to which these strategies have been adopted by intensivists is unknown.

**Methods:**

We developed a six-section questionnaire that covered the drugs used, modalities of drug administration, use of sedation scales and procedural pain scales, use of written local procedures, and targeted objectives of consciousness. In November 2011, the questionnaire was sent to 1,078 intensivists identified from the French ICU Society (SRLF) database.

**Results:**

The questionnaire was returned by 195 intensivists (response rate 18.1%), representing 135 of the 282 ICUs (47.8%) listed in the French ICU society (SRLF) database. The analysis showed that midazolam and sufentanil are the most frequently used hypnotics and opioids, respectively, administered in continuous intravenous (IV) infusions. IV boluses of hypnotics without subsequent continuous IV infusion are used occasionally (in <25% of patients) by 65% of intensivists. Anxiolytic benzodiazepines (e.g., clorazepam, alprazolam), hydroxyzine, and typical neuroleptics, via either an enteral or IV route, are used occasionally by two thirds of respondents. The existence of a written, local sedation management procedure in the ICU is reported by 55% of respondents, 54% of whom declare that they use it routinely. Written local sedation procedures mainly rely on titration of continuous IV hypnotics (90% of the sedation procedures); less frequently, sedation procedures describe alternative approaches to prevent oversedation, including daily interruption of continuous IV hypnotic infusion, hypnotic boluses with no subsequent continuous IV infusion, or the use of nonhypnotic drugs. Among the responding intensivists, 98% consider eye opening, either spontaneously or after light physical stimulation, a reasonable target consciousness level in patients with no severe respiratory failure or intracranial hypertension.

**Conclusions:**

Despite a low individual response rate, the respondents to our survey represent almost half of the ICUs in the French SRLF database. The presence of a written local sedation procedure, a cornerstone of preventing oversedation, is reported by only half of respondents; when present, it is used in for a limited number of patients. Sedation procedures mainly rely on titration of continuous IV hypnotics, but other strategies to limit oversedation also are included in sedation procedures. French intensivists no longer consider severely altered consciousness a sedation objective for most patients.

## Background

Patients who are mechanicallyventilated in the intensive care unit (ICU) commonly receive sedation, either alone or in combination with analgesia, to relieve pain and discomfort and to control agitation and ventilator dyssynchrony. Most sedative drugs have potent hypnotic properties; thus excessive sustained alteration of consciousness is a major side effect of sedation [1]. The main consequence is an increased duration of mechanical ventilation, which is now a common surrogate marker of oversedation. Oversedation also results in increased rates of ventilator-associated pneumonia [2] and ICU-acquired weakness [3].

Different strategies have been proven to reduce oversedation and are recommended by the French ICU Society sedation guidelines in 2007 [4] and more recently by the Society of Critical Care Medicine sedation guidelines in 2013 [5]. These include the targeted titration of continuous intravenous (IV) infusions of hypnotics and daily interruption of continuous IV infusions of hypnotics. The importance of formalizing the sedation strategy in a written, local procedure is also emphasized; the procedure should include repeated measurements of consciousness level on a sedation scale and the detection and treatment of procedural pain. It is unknown whether written local procedures are used and what type of oversedation prevention strategy is currently used in French ICUs.

Alternatives to continuous around-the-clock IV infusions of hypnotics have recently been proposed. These alternatives include short-duration (e.g., 6-h duration) IV infusions of hypnotics [6], repeated IV boluses of hypnotics (with no continuous IV infusion) [7], or the use of nonhypnotic drugs, such as neuroleptics [6,8]. Increasingly the concept of light sedation has emerged, where (once discomfort, dyssynchrony, and agitation have been controlled) patient awakeness and cooperation are promoted, rather than deep alterations of consciousness. It is unknown how often alternatives to continuous IV hypnotic infusion are used in daily practice or what targets of consciousness are currently used among intensivists.

In the present study, we conducted a survey of French ICUs to determine the perceived sedation practices in patients that require mechanical ventilation (invasive ventilation). We investigated the use of continuous IV hypnotics and alternatives to continuous IV hypnotics, the use of local written procedure; the type of strategy used to prevent oversedation, the detection and treatment of procedural pain, and the assessment and objectives of consciousness level. The information provided by this survey may stimulate educational interventions.

## Methods

The questionnaire was developed by three senior intensivists (BDJ, FV, and GP) experienced in sedation of patients with critically illnesses (see Additional file 1). The first of the six sections of the questionnaire summarized the characteristics of the intensivists (experience in ICU, full- or part-time position, description of hospital and ICU). The following sections collected data about the drugs used (midazolam, propofol, nonhypnotic benzodiazepines, hydroxyzine, neuroleptics, opioids), the routes of administration (continuous infusion, IV bolus, enteral route), the use of a sedation scale and Bispectral index (BIS), the use of a pain scale in communicating and non-communicating patients, the use of a written local procedure, and the sedation objective in a patient with no severe respiratory failure or intracranial hypertension. We did not record the use of delirium scales because, despite the uncontroversial prognostic value of delirium in critically ill patients, therapeutic strategies based on delirium assessment, conversely to those based on the use of sedation and pain scales, are sparse and, to our knowledge, their impact on outcome has not been assessed so far. Most of the items in the second set of sections were designed to be answered with a Likert scale based on the following four anchors: “in more than 75% of patients”; “in 25-75% of patients”; “in less than 25% of patients”; and “never.” After data collection and analysis, the anchor labels were transformed to “routinely,” “often,” “occasionally” and “never”, respectively. Each questionnaire item was discussed by six intensivist members of the Epidemiology and Clinical Research Committee of the French ICU Society (BDJ, FV, GP, JA, SL, AG) until no further issue arose regarding educational value, relevance, clarity, and ease of completion.

In November 2011, the survey was emailed to 1,078 intensivists (seniors or assistants, excluding residents) in university- and nonuniversity-affiliated adult ICUs across France. The intensivists were identified from the French ICU Society (SRLF) database. After 1 and 2 weeks, reminders were emailed to non-respondents. We offered no compensation for participation in the survey.

The data are described as the number and percentage or as the median and interquartile range (IQR).

## Results

**Figure 1:**
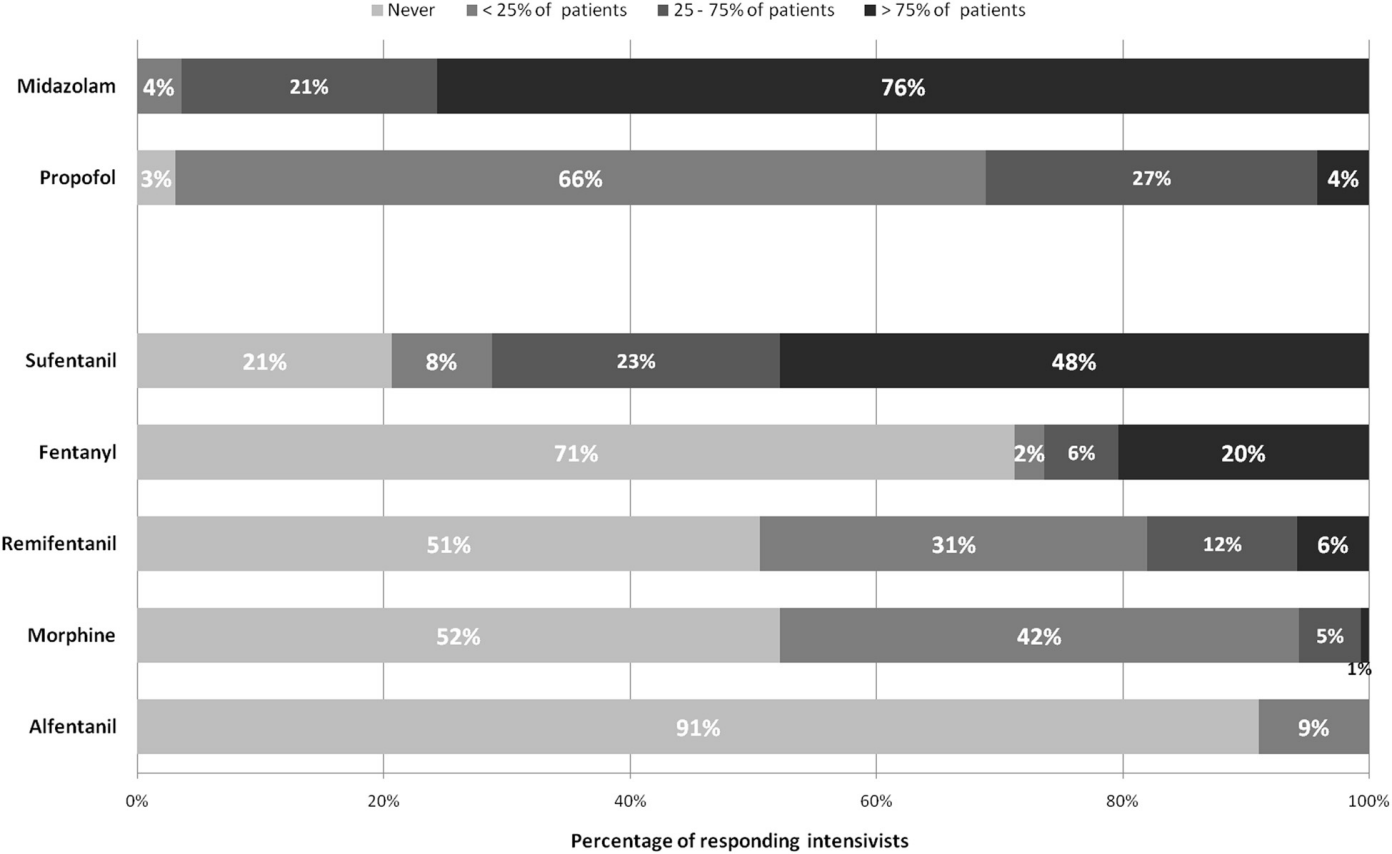
Use of IV continuous infusions of hypnotics and opioids.

The questionnaire was returned by 195 intensivists (response rate 18.1%), representing 135 of the 282 ICUs (47.8%) listed in the French ICU society (SRLF) database. Table 1 reports the main characteristics of the respondents. Notably, 77% of intensivists were full-time senior intensivists, and 66% had more than 10 years experience in the care of critically ill patients. Continuous IV midazolam is used routinely (in >75% of patients) by 76% of the responding intensivists, whereas continuous IV propofol is used only occasionally (in <25% of patients) by 66% of the respondents (Figure 1). Sufentanil is the most frequently used continuous IV opioid, with 48% of the responding intensivists reporting routine use of this opioid (Figure 1). Subcutaneous morphine is used never or occasionally by 52% and 42% of the respondents, respectively.

**Table 1:** Characteristics of the 195 responding intensivists

**Intensivist status**	
Senior intensivist, full time in ICU	77%
Senior intensivist, part-time in ICU	3%
Assistant	10%
Other	11%
Experience in critical care (yr)	
>10	66%
5-10 yrs	19%
2-5	11%
<2	4%
Type of hospital	
University affiliated	33%
Non university affiliated	51%
Private	14%
Other	2%
Type of ICU	
Medico-surgical	74%
Medical	19%
Surgical	6%
Other	2%
ICU activity in 2010	
Number of ICU beds, median (IQR)	12 (10;16)
Number of ICU admissions	
<250	3%
250-500	43%
500-750	33%
750-1000	16%
>1,000	5%
Proportion of patients with mechanical ventilation	
<20%	0%
20-40%	16%
40-60%	44%
60-80%	32%
>80%	7%
Number of physicians (full-time equivalent), median (IQR)	6 (4;7)
Usual patient-to-nurse ratio, median (IQR)	2,5 (2,5;3)

*ICU*, Intensive care unit; *IQR*, Interquartile range.

**Figure 2:**
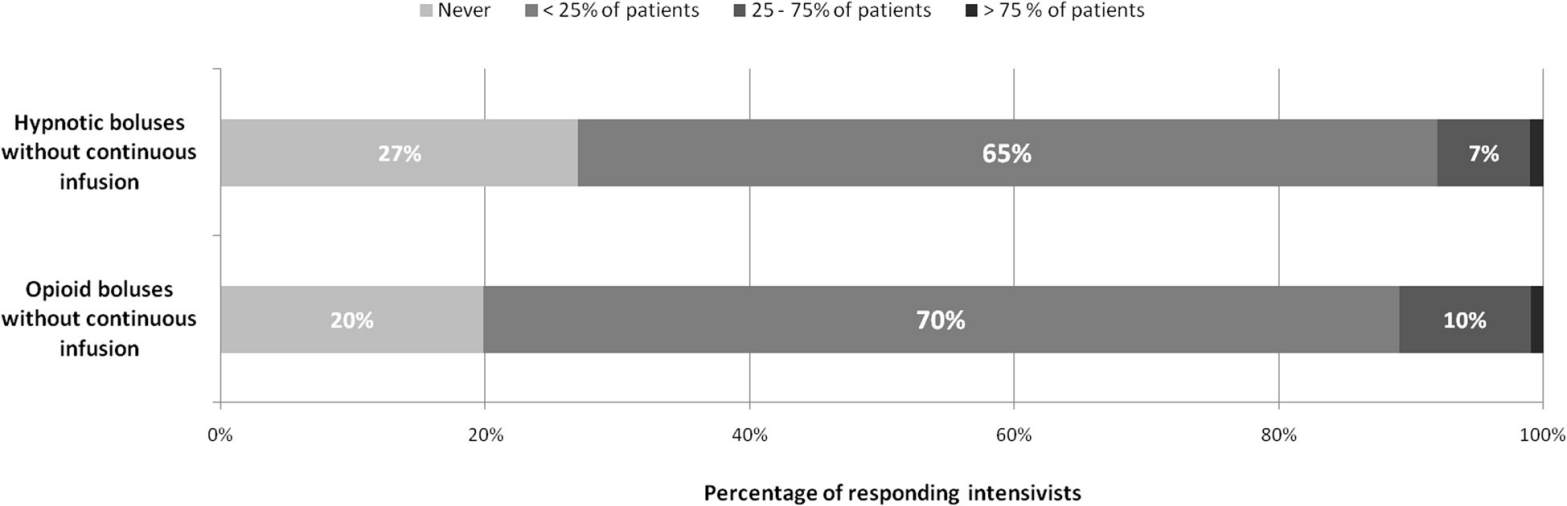
Use of IV hypnotic and opioid boluses without subsequent continuous IV infusion.

**Figure 3:**
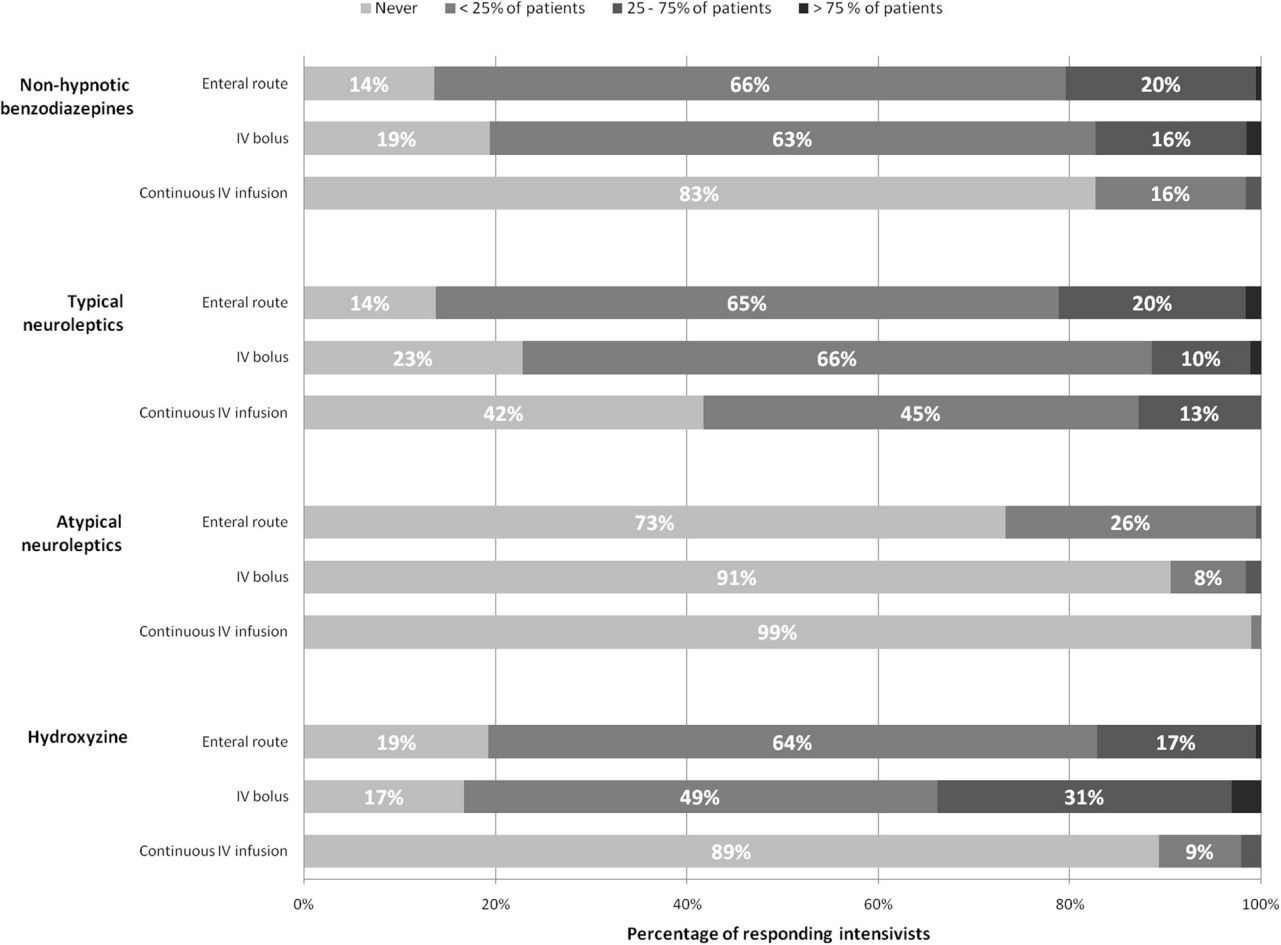
**Use of alternatives to IV hypnotics.** Nonhypnotic benzodiazepines include clorazepam and alprazolam. Typical neuroleptics include haloperidol, lévomépromazine, and cyamemazine. Atypical neuroleptics include loxapine, olanzapine, and risperidone.

A majority of intensivists (65%) report occasional use of IV boluses of hypnotics delivered without subsequent continuous IV infusion (Figure 2). Nonhypnotic benzodiazepines (e.g., clorazepam, alprazolam) delivered via the enteral route and via IV boluses are used occasionally by 66% and 63% of the responding intensivists, respectively (Figure 3). A similar pattern is observed for hydroxyzine and typical neuroleptics (e.g., haloperidol, levomepromazine, cyamemazine). Atypical neuroleptics (e.g., loxapine, olanzapine, risperidone) are almost never used (Figure 3).

**Figure 4:**
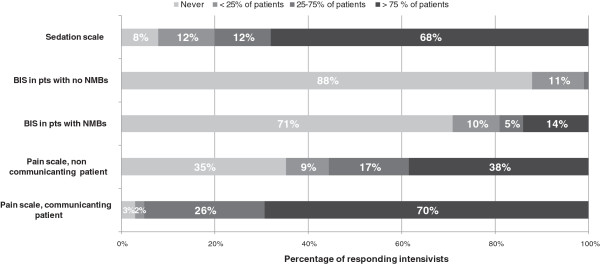
**Use of sedation scales, BIS and pain scales.** NMBs, neuromuscular blockers.

Routine use of a sedation scale is reported by only 68% of responding intensivists (Figure 4). The Ramsay scale and the RASS are used by 50% and 38%, respectively, of the intensivists using a sedation scale. These assessments are primarily performed by nurses (Table 2). The BIS is almost never used, regardless of whether the patient is receiving neuromuscular blockers or not. The routine use of pain scales for assessing communicating patients undergoing potentially painful procedures is reported by 70% of respondents. However, only 38% of respondents report routine pain scale use in noncommunicating patients (Figure 4). The most frequently used pain scales were the Behavioral Pain Scale (BPS) in non-communicating patients (80% of respondents), and analogous scales in communicating patients (98% of respondents); again pain levels are mainly assessed by nurses (Table 2).

**Table 2:** Reported aspects of the use of sedation and pain scales, when used

**Intensivists reporting the use of a scale**	**Sedation scale**		**Pain scale in non-communicating patients during potentially painful procedures**		**Pain scale in communicating patients during potentially painful procedures**	
Scale, no. of intensivists (%)	Ramsay scale	50%	BPS	80%	Analogous scale	98%
	RASS	38%	Locally-designed scale	9%	BPS	9%
	ATICE Scale	8%	Other*	12%	Other*	6%
	SAS	4%				
	Other	9%				
Assessment, no. of intensivists (%)						
By nurses mostly	91%		93%		90%	
By doctors mostly	1%		1%		0%	
By both nurses and doctors	8%		7%		10%	
Frequency, no. of intensivists (%)						
At least every 4 hr	73%					
At least every 12 hr	16%					
At least once a day	10%					

*RASS*, Richmond agitation sedation scale; *ATICE*, Adaptation to intensive care environment; *SAS*, Sedation agitation scale; *BPS*, Behavior pain scale.

Analogous scale, includes visual scale, numerical scale.

*Several intensivists reported the use of 2 scales or more, resulting in total percentage >100%.

**Figure 5:**
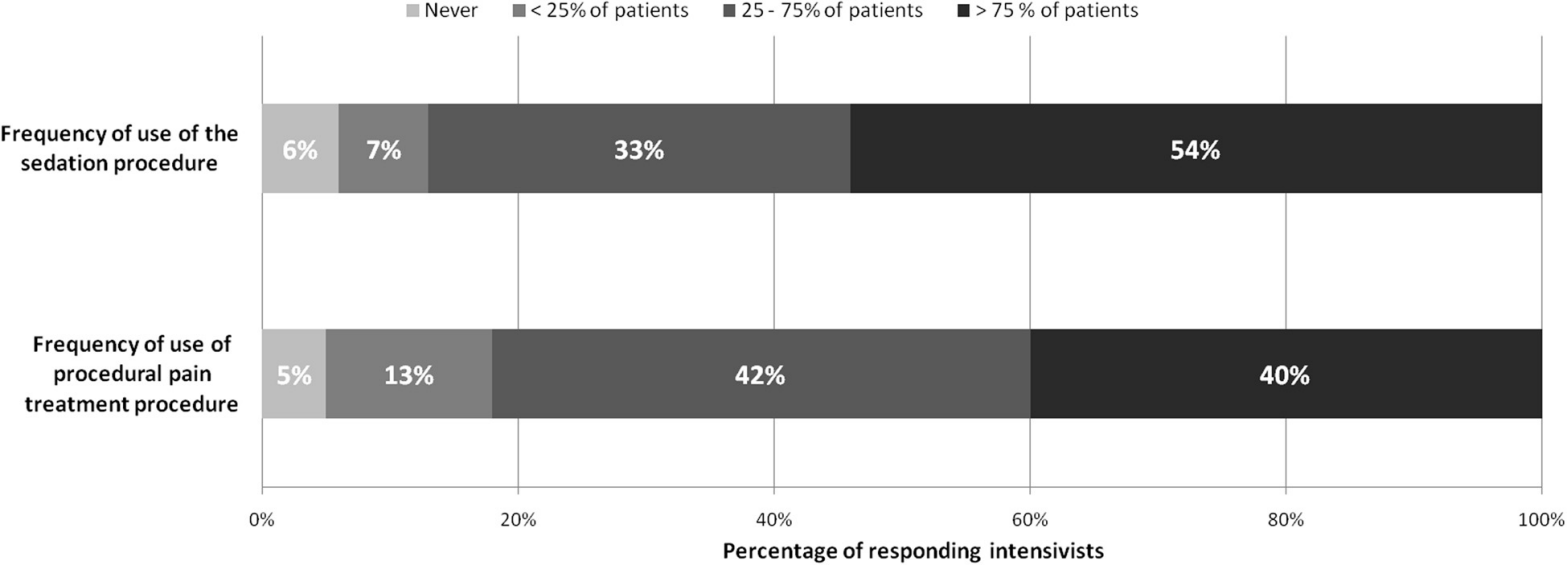
**Use of sedation and procedural pain treatment procedures in ICUs where such procedures exist locally.** The presence of a written local sedation management procedure in the ICU was reported by 55% of the responding intensivists. The presence of a written local pain management procedure in the ICU was reported by 45% of the intensivists (see text).

**Figure 6:**
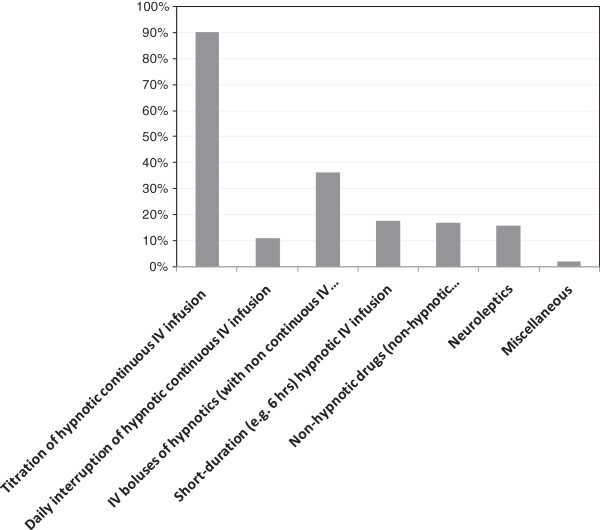
Sedation strategy included in the local, written sedation procedure.

The presence of a written, local sedation, management procedure in the ICU is reported by 55% of responding intensivists. However, in ICUs with these procedures, only 54% of intensivists declare they use it routinely (Figure 5). The presence of a written local pain management procedure in the ICU is reported by 45% of the intensivists. However, in ICUs with these procedures, only 40% of respondents use the procedure routinely. Written, local sedation procedures mainly rely on the titration of continuous IV hypnotics according to patient consciousness and tolerance (90% of the sedation procedures). The use of IV boluses that were not followed by continuous infusion also is reported in 30% of procedures. Other strategies, including daily interruption of continuous IV hypnotics, are used much less frequently (Figure 6).

Among the responding intensivists, 61% consider spontaneous eye opening to be a reasonable consciousness target level in patients with no severe acute respiratory distress syndrome (ARDS) or intracranial hypertension (ICH); 37% consider eye opening after a light physical stimulation to be a reasonable target. Eye opening to a strong noxious stimulation is considered a reasonable consciousness target by 2% of responders, whereas no respondents consider no eye opening, whatever the stimulation, a reasonable target.

## Discussion

In this survey, midazolam and sufentanil appear as the most frequently used hypnotic and opioid, respectively, administered in continuous IV infusions. IV boluses of hypnotics without subsequent continuous IV infusion, anxiolytic benzodiazepines (e.g., clorazepam, alprazolam), hydroxyzine, and typical neuroleptics, via either an enteral or IV route, are used occasionally by two thirds of respondents. The existence of a written, local sedation management procedure in the ICU (mainly relying on continuous IV hypnotic titration) is reported by 55% of respondents, 54% of whom declare that they use it routinely. Among the responding intensivists, 98% consider eye opening, either spontaneously or after light physical stimulation, a reasonable target consciousness level in patients with no severe respiratory failure or intracranial hypertension.

Despite the strong recommendations of the 2007 French [4] and 2013 U.S. [5] Consensus Conferences to monitor sedation using clinical scales, and the large number of scales currently available and validated for the ICU setting, less than 70% of the responding intensivists report the routine use of a sedation scale. Routine objective detection of procedural pain in communicating patients is reported by a similar number of respondents (70%), but that frequency contrasts sharply with the further lower rate of detecting pain in non-communicating patients, as only 38% of respondents report the routine use of a pain scale in these patients. This likely reflects the common, persistent belief that noncommunicating patients, whose consciousness is frequently altered, are unlikely to feel pain. Furthermore, in a highly technically sophisticated environment dedicated to the treatment of life-threatening organ failures, pain detection might still not be considered a priority. Yet, validated, simple-to-use tools exist, including the BPS [9]. The detection of pain and assessment of the response to analgesics have been shown to have favorable impact on outcomes for patients in the ICU, including those with a noncommunicating phase during the ICU stay [10].

Despite numerous trials showing that written sedation algorithms beneficially affect important outcome markers of oversedation, including mechanical ventilation duration [2,11-13], only 55% of the responding intensivists have a written sedation procedure in their ICU. Furthermore, when a written sedation procedure exists, it is not used routinely by nearly 50% of respondents. There are numerous barriers to the implementation of written sedation procedures, including insufficient education programs, understaffing (in particular, there is a high patient-to-nurse ratio in most French ICUs) and the reluctance of intensivists to transfer sedation management to nurses, which is an integral part of most published algorithms. It also should be acknowledged that written sedation procedures might not apply to some specific ICU patients, particularly those with severe brain injury or those in whom treatment withdrawal has been decided.

Titration of continuous IV infusion of hypnotics is by far the most common method used to prevent oversedation in French ICUs. This is in accordance with the 2007 French Consensus conference guidelines, which included a frame for hypnotics and morphinics use based on continuous titration, whereas the use of daily interruption of continuous IV infusions of hypnotics was not addressed [4]. Inversely, a recent survey showed that 80% of U.S. hospitals use daily interruption of sedatives in mechanically ventilated patients [14]. Although both continuous titration [2,11,12] and daily interruption of sedatives [15] have shown a significant beneficial effect on mechanical ventilation duration, they have not been formally compared. However, a recent randomized trial in North America showed that combining daily interruption of sedatives and continuous titration did not improve outcomes compared to continuous titration alone but was associated with increased nurse workload [16].

The IV bolus of a hypnotic with no subsequent continuous IV infusion is present in more than 30% of written sedation procedures. In a randomized trial of repeated IV boluses of midazolam with a goal of 1–2 on the Ramsay scale compared to a continuous IV infusion with a Ramsay goal of 3–4, the light sedation strategy with repeated IV boluses revealed feasible and safe and was associated with significantly shorter mechanical ventilation duration and ICU stay and no long-term, adverse cognitive, or psychological impact [7]. This approach therefore might be an interesting alternative to the continuous IV infusion of hypnotics, particularly in patients with moderatelyaltered tolerance to the ICU environment.

The infrequent incorporation of nonhypnotic anxiolytic benzodiazepines, hydroxyzine, or neuroleptics in sedation procedures (15% of the local procedures) contrasts with the high percentage of intensivists (approximately 65%) that report occasional use. This suggests that some aspects of daily practice like care of agitated patients or weaning from IV hypnotics remain to be captured by local procedures. Neuroleptics have been proposed as first-line drugs for controlling agitation, discomfort, and delirium [6] and ventilator dyssynchrony [8]. However, their sparing effect on hypnotic use requires further investigation.

Finally, we found that more than 60% of respondents consider spontaneous eye opening a reasonable consciousness target in patients with no severe ARDS or brain injury; 35% of intensivists targeted eye opening to slight verbal or nociceptive stimulus. After several editorials in the 2000th pledging the need for lighter sedation objectives and cooperative sedation for ICU patients [17,18], our finding reflects the considerable shift in the paradigm of sedation practice among intensivists during the past decade.

This survey has several limitations. First, the low response rate of 18.1% might question the generalizability of the results. However, compared to postal mail surveys, email surveys, commonly allowing for larger target population, frequently have lower response rates [19-21]. The response rate of a recent large email survey with questionnaires sent to 6,227 gastroenterologists listed in the American College of Gastroenterology database was 9.5% [22]. Interestingly, the number of respondents in our survey is comparable to the 273 respondents to a survey of sedation practices over Canada in 2006 sent by postal mail to a relatively low number of critical care physicians (448), resulting in a high response rate of 60% [23]. Of note, the 195 respondents to our survey represent almost half of the ICUs listed in the French ICU society (SRLF) database. Furthermore, the respondents in our survey represent a broad range of ICU characteristics (university and nonuniversity hospitals; medical, surgical, and mixed ICUs; large and small ICUs; and various annual ICU admission rates); additionally, the demographic pattern is similar to that of previous surveys of sedation practices in French ICUs [24,25]. A second limitation is that results of practice surveys might differ from the true bedside practice, mainly because perception is inherently subjective. Our study therefore differs from the observational study of sedation practices conducted in 2007 with patient-based data collected in 44 French ICUs [26]. However, our aim in this study was to address the perception of sedation practices among intensivists, not the actual practices.

## Conclusions

Despite a low individual response rate, the respondents to our survey represent almost half of the ICUs in the French SRLF database. This survey revealed that the written sedation procedure, a cornerstone for the prevention of oversedation, is present in only 50% of respondents’ ICUs. Furthermore, we found that when a written sedation procedure exists, it is used in only a limited number of patients. In addition, procedural pain is frequently detected in communicating patients, but not in noncommunicating patients. The use of procedures for detecting and treating procedural pain also is limited. Educational measures are warranted to improve these findings. Our study also revealed that several alternatives to the common continuous IV hypnotic infusions, including repeated IV hypnotic boluses or the use of nonhypnotic drugs, can be judiciously included in local sedation procedures to limit oversedation. Finally, French intensivists no longer consider severely altered consciousness an objective of sedation for most patients.

## Appendix

The members of the writing committee for the SRLF Trial group are as follows:

De Jonghe, MD, Réanimation Médico-Chirurgicale, Centre Hospitalier de Poissy, 10 rue du champ gaillard, Poissy 78300, France ; Epidemiology and Clinical Research Committee of the French ICU Society (SRLF)

François Vincent, MD, Réanimation Médico-Chirurgicale, Centre Hospitalo-Universitaire Avicenne, 125 route de Stalingrad, Bobigny 93009, France; Epidemiology and Clinical Research Committee of the French ICU Society (SRLF)

Gaetan Plantefeve, MD, Réanimation Polyvalente, Centre Hospitalier d’Argenteuil, 69 rue du Lieutenant Colonel Prudhon, Argenteuil 95107, France; Epidemiology and Clinical Research Committee of the French ICU Society (SRLF)

Jerome Aboab, MD, Réanimation Médicale, Centre Hospitalo-Universitaire Raymond Poincaré, 104 boulevard Raymond Poincaré, Garches 92380, France; Epidemiology and Clinical Research Committee of the French ICU Society (SRLF)

Stéphane Legriel, MD, Réanimation, Centre Hospitalier de Versailles, 177 rue de Versailles, Le Chesnay 78157, France; Epidemiology and Clinical Research Committee of the French ICU Society (SRLF)

Antoine Gros, MD, Réanimation, Centre Hospitalier de Versailles, 177 rue de Versailles, Le Chesnay 78157, France; Epidemiology and Clinical Research Committee of the French ICU Society (SRLF)

Elie Azoulay, MD, PhD, Réanimation Médicale, Centre Hospitalo-Universitaire Saint-Louis, 1 avenue Claude Vellefaux, Paris 75475, France

Jean Reignier, MD, Réanimation Polyvalente, Centre Hospitalier Les Oudairies, boulevard Stéphane Moreau, La Roche-sur-Yon 85925, France

Djillali Annane, MD, PhD, Réanimation Médicale, Centre Hospitalo-Universitaire Raymond Poincaré, 104 boulevard Raymond Poincaré, Garches 92380, France; Board Chairman, French ICU Society (SRLF)

## Abbreviations

ICU: Intensive care unit; IV: Intravenous.

## Competing interests

The authors declare that they have no competing interests.

## Authors’ contributions

BDJ participated in the conception and design of the questionnaire and study, analysis and interpretation of the data, and drafting the manuscript. FV, GP, SL, AG, and JA participated in the design of the questionnaire and study, interpretation of the data, and critical revision of the manuscript. EA, JR, and DA participated in the design of the study, interpretation of the data, and critical revision of the manuscript. All authors have given final approval of the final manuscript.

## Supplementary Material

Additional file 1 Questionnaire used in the study. Click here for file

## **The French ICU society (SRLF) trial group**

The members of the writing committee for the SRLF trial group (listed in the Appendix) assume responsibility for the content of this article.

## References

[B1] Patel SB Kress JP Sedation and analgesia in the mechanically ventilated patient Am J Respir Crit Care Med 2012 3 486 497 10.1164/rccm.201102-0273CI 22016443

[B2] Quenot JP Ladoire S Devoucoux F Doise JM Cailliod R Cunin N Aube H Blettery B Charles PE Effect of a nurse-implemented sedation protocol on the incidence of ventilator-associated pneumonia Crit Care Med 2007 3 2031 2036 10.1097/01.ccm.0000282733.83089.4d 17855817

[B3] De Jonghe B Lacherade JC Sharshar T Outin H Intensive care unit-acquired weakness: risk factors and prevention Crit Care Med 2009 3 S309 S315 20046115 10.1097/CCM.0b013e3181b6e64c

[B4] Sauder P Andreoletti M Cambonie G Capellier G Feissel M Gall O Goldran-Toledano D Kierzek G Mateo J Mentec H Sedation and analgesia in intensive care (with the exception of new-born babies) Ann Fr Anesth Reanim 2008 3 541 551 10.1016/j.annfar.2008.04.021 18579339

[B5] Barr J Fraser GL Puntillo K Ely EW Gelinas C Dasta JF Davidson JE Devlin JW Kress JP Joffe AM Clinical practice guidelines for the management of pain, agitation, and delirium in adult patients in the intensive care unit Crit Care Med 2013 3 263 306 23269131 10.1097/CCM.0b013e3182783b72

[B6] Strom T Martinussen T Toft P A protocol of no sedation for critically ill patients receiving mechanical ventilation: a randomised trial Lancet 2010 3 475 480 10.1016/S0140-6736(09)62072-9 20116842

[B7] Treggiari MM Romand JA Yanez ND Deem SA Goldberg J Hudson L Heidegger CP Weiss NS Randomized trial of light versus deep sedation on mental health after critical illness Crit Care Med 2009 3 2527 2534 10.1097/CCM.0b013e3181a5689f 19602975

[B8] Sztrymf B Chevrel G Bertrand F Margetis D Hurel D Ricard JD Dreyfuss D Beneficial effects of loxapine on agitation and breathing patterns during weaning from mechanical ventilation Crit Care 2010 3 R86 10.1186/cc9015 20459867 PMC2911718

[B9] Payen JF Bru O Bosson JL Lagrasta A Novel E Deschaux I Lavagne P Jacquot C Assessing pain in critically ill sedated patients by using a behavioral pain scale Crit Care Med 2001 3 2258 2263 10.1097/00003246-200112000-00004 11801819

[B10] Chanques G Jaber S Barbotte E Violet S Sebbane M Perrigault PF Mann C Lefrant JY Eledjam JJ Impact of systematic evaluation of pain and agitation in an intensive care unit Crit Care Med 2006 3 1691 1699 10.1097/01.CCM.0000218416.62457.56 16625136

[B11] Brook AD Ahrens TS Schaiff R Prentice D Sherman G Shannon W Kollef MH Effect of a nursing-implemented sedation protocol on the duration of mechanical ventilation Crit Care Med 1999 3 2609 2615 10.1097/00003246-199912000-00001 10628598

[B12] De Jonghe B Bastuji-Garin S Fangio P Lacherade JC Jabot J Appere-De-Vecchi C Rocha N Outin H Sedation algorithm in critically ill patients without acute brain injury Crit Care Med 2005 3 120 127 10.1097/01.CCM.0000150268.04228.68 15644658

[B13] Brattebo G Hofoss D Flaatten H Muri AK Gjerde S Plsek PE Effect of a scoring system and protocol for sedation on duration of patients’ need for ventilator support in a surgical intensive care unit BMJ 2002 3 1386 1389 10.1136/bmj.324.7350.1386 12052813 PMC1123333

[B14] Miller MA Krein SL Saint S Kahn JM Iwashyna TJ Organisational characteristics associated with the use of daily interruption of sedation in US hospitals: a national study BMJ Qual Saf 2012 3 145 151 10.1136/bmjqs-2011-000233 21949434 PMC4005254

[B15] Kress J Pohlman A O’Connor M Hall J Daily interruption of sedative infusions in critically ill patients undergoing mechanical ventilation N Engl J Med 2000 3 1471 1477 10.1056/NEJM200005183422002 10816184

[B16] Mehta S Burry L Cook D Fergusson D Steinberg M Granton J Herridge M Ferguson N Devlin J Tanios M Daily sedation interruption in mechanically ventilated critically ill patients cared for with a sedation protocol: a randomized controlled trial JAMA 2012 3 1985 1992 10.1001/jama.2012.13872 23180503

[B17] Heffner JE A wake-up call in the intensive care unit N Engl J Med 2000 3 1520 1522 10.1056/NEJM200005183422011 10816193

[B18] Hansen-Flaschen J Improving patient tolerance of mechanical ventilation. Challenges ahead Crit Care Clin 1994 3 659 671 8000919

[B19] VanDenKerkhof EG Parlow JL Goldstein DH Milne B In Canada, anesthesiologists are less likely to respond to an electronic, compared to a paper questionnaire Can J Anaesth 2004 3 449 454 10.1007/BF03018307 15128630

[B20] Leece P Bhandari M Sprague S Swiontkowski MF Schemitsch EH Tornetta P Devereaux PJ Guyatt GH Internet versus mailed questionnaires: a controlled comparison (2) J Med Internet Res 2004 3 e39 10.2196/jmir.6.4.e39 15631963 PMC1550620

[B21] Crouch S Robinson P Pitts M A comparison of general practitioner response rates to electronic and postal surveys in the setting of the National STI Prevention Program Aust N Z J Public Health 2011 3 187 189 10.1111/j.1753-6405.2011.00687.x 21463418

[B22] Tinsley A Naymagon S Trindade AJ Sachar DB Sands BE Ullman TA A survey of current practice of venous thromboembolism prophylaxis in hospitalized inflammatory bowel disease patients in the United States J Clin Gastroenterol 2013 3 e1 e6 10.1097/MCG.0b013e31824c0dea 22476043

[B23] Mehta S Burry L Fischer S Martinez-Motta JC Hallett D Bowman D Wong C Meade MO Stewart TE Cook DJ Canadian survey of the use of sedatives, analgesics, and neuromuscular blocking agents in critically ill patients Crit Care Med 2006 3 374 380 10.1097/01.CCM.0000196830.61965.F1 16424717

[B24] Constantin JM Chanques G De Jonghe B Sanchez P Mantz J Payen JF Sztark F Richebe P Lagneau F Capdevila X La sédation-analgésie au quotidien : enquête de pratiqueauprès de 218 services de réanimation en France Ann Fr Anesth Reanim 2010 3 339 346 10.1016/j.annfar.2010.01.014 20392591

[B25] De Jonghe B Constantin JM Chanques G Capdevila X Lefrant JY Outin H Mantz J Physical restraint in mechanically ventilated ICU patients: a French practice survey Intensive Care Med 2012 10.1007/s00134-00012-02715-00139 23064500

[B26] Payen JF Chanques G Mantz J Hercule C Auriant I Leguillou JL Binhas M Genty C Rolland C Bosson JL Current practices in sedation and analgesia for mechanically ventilated critically Ill patients: a prospective multicenter patient-based study Anesthesiology 2007 3 687 695 10.1097/01.anes.0000264747.09017.da 17413906

