# Supplementary material for: Sedation in French intensive care units: a survey of clinical practice
Source: Ann Intensive Care. 2013 Aug 9;3:24. doi: 10.1186/2110-5820-3-24 (PMC3751696; doi:10.1186/2110-5820-3-24)
Supplement: Additional file 1 — Questionnaire used in the study. [file 2110-5820-3-24-S1.docx]

**Sedation in French Intensive Care Units: a Survey of Clinical Practice**

The French ICU Society (SRLF) Trial Group

**Additional file 1. Questionnaire used in the study**

**Section 1. Respondent characteristics**

| **Intensivist status** |  |
| --- | --- |
| Senior intensivist, full time in ICU | □ |
| Senior intensivist, part-time in ICU | □ |
| Assistant | □ |
| Other | □ _ _ _ _ _ _ |
| **Experience in critical care** |  |
| > 10 yrs | □ |
| 5 to 10 yrs | □ |
| 2 to 5 yrs | □ |
| < 2 yrs | □ |
| **Type of hospital** |  |
| University affiliated | □ |
| Non university affiliated | □ |
| Private | □ |
| Other | □ _ _ _ _ _ _ |
| **Type of ICU** |  |
| Medical-surgical ICU | □ |
| Medical ICU | □ |
| Surgical ICU | □ |
| Other (please specify) | □ _ _ _ _ _ _ |
| **ICU activity in 2010** |  |
| Number of ICU beds | _ _ |
| Number of ICU admissions |  |
| < 250 | □ |
| 250-500 | □ |
| 500-750 | □ |
| 750-1000 | □ |
| >1000 | □ |
| Proportion of patients with mechanical ventilation |  |
| < 20% | □ |
| 20-40% | □ |
| 40-60% | □ |
| 60-80% | □ |
| >80% | □ |
| Number of full-time physicians | _ _ |
| Usual patient-to-nurse ratio | _ _,_ |

**Section 2. Sedative and analgesic drugs used**

**2.1 Which continuous IV hypnotics do you use?**

|  | Never | In < 25% of patients | In 25 to 75% of patients | In > 75% of patients |
| --- | --- | --- | --- | --- |
| Midazolam |  |  |  |  |
| Propofol |  |  |  |  |

**2.2 Which continuous IV opioids do you use?**

|  | Never | In < 25% of patients | In 25 to 75% of patients | In > 75% of patients |
| --- | --- | --- | --- | --- |
| Morphine IV |  |  |  |  |
| Fentanyl IV |  |  |  |  |
| Sufentanil IV |  |  |  |  |
| Remifentanil IV |  |  |  |  |
| Alfentanil IV |  |  |  |  |

**2.3 Do you use subcutaneous morphine ?**

|  | Never | In < 25% of patients | In 25 to 75% of patients | In > 75% of patients |
| --- | --- | --- | --- | --- |
|  |  |  |  |  |

**2.4 Do you use hypnotics (among those listed above) boluses without continuous infusion?**

|  | Never | In < 25% of patients | In 25 to 75% of patients | In > 75% of patients |
| --- | --- | --- | --- | --- |
|  |  |  |  |  |

**2.5 Do you use opioids (among those listed above) boluses without continuous infusion ?**

|  | Never | In < 25% of patients | In 25 to 75% of patients | In > 75% of patients |
| --- | --- | --- | --- | --- |
|  |  |  |  |  |

**2.6 Do you use the following analgesics?**

|  | Never | In < 25% of patients | In 25 to 75% of patients | In > 75% of patients |
| --- | --- | --- | --- | --- |
| Paracetamol |  |  |  |  |
| Nefopam |  |  |  |  |
| Nalbuphine, tramadadol, buprenorphine… |  |  |  |  |

**2.7 Do you use the following drugs?**

|  | Never | In < 25% of patients | In 25 to 75% of patients | In > 75% of patients |
| --- | --- | --- | --- | --- |
| Ketamine |  |  |  |  |
| Clonidine |  |  |  |  |
| Halogenated gazes with a conserving device |  |  |  |  |
| Transdermal nicotine in smoker patients* |  |  |  |  |

**2.8 Do you use anxiolytic (non-hypnotic) benzodiazepines, including clorazepam, alprazolam…?**

|  | Never | In < 25% of patients | In 25 to 75% of patients | In > 75% of patients |
| --- | --- | --- | --- | --- |
| IV boluses (without continuous IV infusion) |  |  |  |  |
| Continuous IV infusion |  |  |  |  |
| Enteral route |  |  |  |  |

**2.9 Do you use neuroleptics (typical neuroleptics), including haloperidol, levomepromazine, loxapine…?**

|  | Never | In < 25% of patients | In 25 to 75% of patients | In > 75% of patients |
| --- | --- | --- | --- | --- |
| IV boluses (without continuous IV infusion) |  |  |  |  |
| Continuous IV infusion |  |  |  |  |
| Enteral route |  |  |  |  |

**2.10 Do you use atypical neuroleptics, including olanzapine, risperdone…?**

|  | Never | In < 25% of patients | In 25 to 75% of patients | In > 75% of patients |
| --- | --- | --- | --- | --- |
| IV boluses (without continuous IV infusion) |  |  |  |  |
| Continuous IV infusion |  |  |  |  |
| Enteral route |  |  |  |  |

**2.11 Do you use hydroxizine?**

|  | Never | In < 25% of patients | In 25 to 75% of patients | In > 75% of patients |
| --- | --- | --- | --- | --- |
| IV boluses (without continuous IV infusion) |  |  |  |  |
| Continuous IV infusion |  |  |  |  |
| Enteral route |  |  |  |  |

**Section 3. Assessment of sedation depth**

**3.1 In what percentage of patients receiving sedation do you use a clinical sedation scale?**

|  | Never | In < 25% of patients | In 25 to 75% of patients | In > 75% of patients |
| --- | --- | --- | --- | --- |
|  |  |  |  |  |

**3.2 If your answer was not Never, what scale do you use?**

- Ramsay
- RASS
- ATICE
- SAS
- Other

**3.3 How frequently do you use this scale ?**

- At least every 4 hours
- At least every 12 hours
- At least once a day

**3.4 Who is assessing the patient based on the sedation scale?**

- Doctors mostly
- Nurses mostly
- By doctors and nurses in a rather similar proportion

**3.5 Do you use the BIS to monitor sedation depth in patients with no neuromuscular blockers?**

|  | Never | In < 25% of patients | In 25 to 75% of patients | In > 75% of patients |
| --- | --- | --- | --- | --- |
|  |  |  |  |  |

**3.6 Do you use the BIS to monitor sedation depth in patients with neuromuscular blockers?**

|  | Never | In < 25% of patients | In 25 to 75% of patients | In > 75% of patients |
| --- | --- | --- | --- | --- |
|  |  |  |  |  |

**Section 4. Procedural pain assessment**

**4.1 Do you use a pain scale to assess procedural pain in non communicating patients?**

|  | Never | In < 25% of patients | In 25 to 75% of patients | In > 75% of patients |
| --- | --- | --- | --- | --- |
|  |  |  |  |  |

**4.2 If your answer was not Never, what scale do you use?**

- BPS
- Other

**4.3 Who is assessing the patient based on the pain scale?**

- Doctors mostly
- Nurses mostly
- By doctors and nurses in a rather similar proportion

**4.4 Do you use a pain scale to assess procedural pain in communicating patients?**

|  | Never | In < 25% of patients | In 25 to 75% of patients | In > 75% of patients |
| --- | --- | --- | --- | --- |
|  |  |  |  |  |

**4.5 If your answer was not Never, what scale do you use?**

- BPS
- Visual analogous scales, or similar scales
- Other

**4.6 Who is assessing the patient based on the pain scale?**

- Doctors mostly
- Nurses mostly
- By doctors and nurses in a rather similar proportion

**Section 5. Local sedation and procedural pain procedures**

**5.1 Is there in your ICU a written procedure indicating how to adapt sedative dosages according to the patient clinical condition?**

- Yes
- No

**5.2 If your answer was Yes, in what percentage of patients receiving sedation do you use this procedure?**

|  | Never | In < 25% of patients | In 25 to 75% of patients | In > 75% of patients |
| --- | --- | --- | --- | --- |
|  |  |  |  |  |

**5.3 Does the procedure include (multiple choice question):**

- Repeated daily hypnotic titrations?
- Daily interruptions of the continuous IV hypnotic infusion?
- Bolus IV hypnotic injections with no subsequent continuous IV infusion?
- Short duration (a few hours) IV continuous hypnotic infusions?
- The use of anxiolytic (non-hypnotic) benzodiazepines (e.g. clorazepam) or hydroxyzine?
- The use of neuroleptics?

**5.4 Who is doing the dosage changes?**

- Doctors mostly
- Nurses mostly
- By doctors and nurses in a rather similar proportion

**5.5 Is there in your ICU a written procedure indicating to detect and prevent procedural pain?**

- Yes
- No

**5.6 If your answer was Yes, in what percentage of patients do you use this procedure?**

|  | Never | In < 25% of patients | In 25 to 75% of patients | In > 75% of patients |
| --- | --- | --- | --- | --- |
|  |  |  |  |  |

**Section 6. Sedation objective**

**In a mechanically ventilated patient with no severe respiratory distress or intracranial hypertension, at which consciousness level would you try to maintain your patient?**

- Patient awake (eyes open spontaneously)
- Eye opening to voice or light noxious stimulation
- Eye opening to a strong noxious stimulation
- No eye opening, whatever the stimulation
